# Prediction of Fatigue Crack Growth in Gas Turbine Engine Blades Using Acoustic Emission

**DOI:** 10.3390/s18051321

**Published:** 2018-04-25

**Authors:** Zhiheng Zhang, Guoan Yang, Kun Hu

**Affiliations:** College of Mechanical and Electrical Engineering, Beijing University of Chemical Technology, Beijing 100029, China; 2016210258@mail.buct.edu.cn (Z.Z.); 2016200640@mail.buct.edu.cn (K.H.)

**Keywords:** acoustic emission, gas turbine engine blades, fatigue life prediction, fatigue crack propagation

## Abstract

Fatigue failure is the main type of failure that occurs in gas turbine engine blades and an online monitoring method for detecting fatigue cracks in blades is urgently needed. Therefore, in this present study, we propose the use of acoustic emission (AE) monitoring for the online identification of the blade status. Experiments on fatigue crack propagation based on the AE monitoring of gas turbine engine blades and TC11 titanium alloy plates were conducted. The relationship between the cumulative AE hits and the fatigue crack length was established, before a method of using the AE parameters to determine the crack propagation stage was proposed. A method for predicting the degree of crack propagation and residual fatigue life based on the AE energy was obtained. The results provide a new method for the online monitoring of cracks in the gas turbine engine blade.

## 1. Introduction

Gas turbine engines, which are known as the “heart” of aircrafts, are a type of large-scale equipment that require high reliability and extremely sophisticated technology. Low-pressure compressor blades are one of the key components of gas turbine engines and their main function is to convert energy [1]. The blades operate in a very harsh environment. During each flight, they are subjected to the combined effects of high- and low-cycle loads, including centrifugal tensile stress and centrifugal bending moments, aerodynamic stress and aerodynamic bending moments, thermal stress and vibration as well as alternating stress [2]. In addition, because the compressor blades are located in the front of the gas turbine engine, they are vulnerable to atmospheric corrosion and the impact of dust, sand, birds and other foreign objects in the high-speed intake airflow [3]. Stress concentration, residual stress, structural changes and possible micro-cracks caused by environmental corrosion and foreign object impact greatly reduce the fatigue strength of the blades under cyclic loading [4], which results in fatigue fractures in the blades. Research on the structural failure of engines has shown that high-cycle fatigue failure is the primary failure mode. About 25% of gas turbine engine accidents are caused by high-cycle fatigue [5].

Non-destructive testing of the blades is frequently required for gas turbine engine maintenance in order to prevent serious accidents caused by fatigue damage of the blades. These testing methods include ultrasonic detection, radiographic inspection and endoscopy [6,7,8]. However, these methods require empirical judgment by human operators using manuals and can be easily influenced by multiple factors [9]. In addition, the testing procedures are time-consuming and labor-intensive, resulting in high maintenance costs. More accurate information on the blade status and an improvement in condition-based maintenance are important for improving the economics of operating gas turbine engines [10], which requires sound condition monitoring technology. At present, the most widely used gas turbine engine condition monitoring is vibration monitoring [11]. However, it is difficult to transmit the vibration information generated by the blade cracks to the vibration sensors due to the complex structure of gas turbine engines. In addition, vibration monitoring only monitors the vibration intensity but cannot identify the initial and early faults [12]. Acoustic emission (AE) testing has become an accepted, suitable and effective non-destructive technique to investigate and evaluate the failure processes of different structural components [13]. The main advantage of AE over the other condition monitoring techniques is that the detected AE signals can be used to characterize the different damage mechanisms [14]. In addition, the AE sensors that are placed on the surface of the gas turbine engine casing receive the crack signal from the blades. Therefore, in this present study, we propose the application of AE monitoring technology for the online identification of the blade state.

AE describes the phenomenon of materials releasing strain energy in the form of transient elastic waves due to plastic deformation, crack formation and crack expansion [15]. AE technology has been widely used and studied in the aerospace field. American Northrup Grumman Corporation monitored the damage of aircraft structures with AE equipment during aircraft fatigue tests and monitored the entire process of crack formation and propagation with AE technology [16]. Paget [17] and Feng [18] both used AE technology for the damage detection during full-scale static strength tests conducted on aircraft, with the real-time monitoring of locations that were poorly accessible and had a high stress concentration being detailed by the AE technique. Scala [19], Pullin [20] and Awerbuch [21] investigated the fatigue of the aircraft fuselage, landing gear and other materials using AE and they demonstrated that AE is a promising technique for non-destructive evaluation of complex structures on the aircraft. Bhuiyan and Giurgiutiu performed in situ acoustic emission fatigue experiments on aircraft grade aluminum material and grouped the AE signals by waveform analysis to explain the complex phenomenon of metal fatigue [22,23,24]. Urbach demonstrated the possibility of assessing fatigue damage of blades by using the acoustic emission method based on a series of researches focusing on the process of factory tests [25]. In addition, many researchers have investigated the AE characteristics of metal materials, such as steel and magnesium–aluminum alloys, during fatigue crack propagation [26,27,28].

In this study, the AE technique was utilized to monitor the fatigue crack growth of the gas turbine engine low-pressure compressor rotor blades and TC11 titanium alloy plates. The AE signals of the TC11 titanium alloy during the propagation of fatigue cracks were obtained and the signals of the two specimens were very similar. The relationship between the cumulative AE hits and the fatigue crack length was established, before a method of using the AE parameters to determine the crack propagation stage was proposed. Based on the fracture mechanics and the experimental results, a mathematical relationship between the AE energy rate and crack growth rate was developed to predict the crack extension rate of the blade material and the residual fatigue life of the blades. The results provide a new method for the online monitoring of gas turbine engine blade cracks.

## 2. Theoretical Model for Life Prediction

In the fatigue study of materials, according to the theory of elastic fracture mechanics, the relationship between the fatigue crack growth rate and the stress intensity factor is usually expressed by the semi-empirical Paris equation [29]:

(1) $$\frac{da}{dN}=C{\left(\Delta K\right)}^{m},$$

where *a* is the crack length; *N* is the number of loading cycles (therefore, (*da/dN*) is the crack growth rate); ΔK is the stress intensity range, with $\Delta K=K_{max}-K_{min}$; and *C* and *m* are the constants determined by the material.

**Figure 1:**
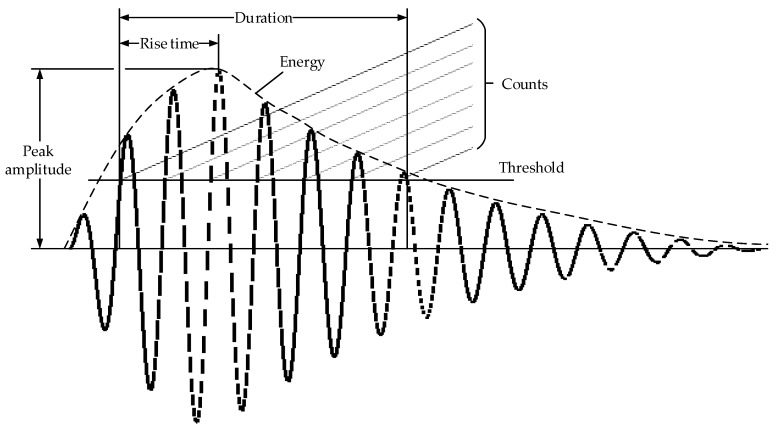
Parameters reflecting an AE waveform [30].

In the AE signal processing methods, simplified characteristic parameters are often used to represent the AE signals. Commonly used parameters include the peak amplitude, energy, counts, duration and rise time [30,31,32], which are shown in Figure 1.

In the field of fatigue crack research using AE, many researchers have tried to establish a relationship between the AE parameters and the behavior of the material [33,34,35]. Harris and Dunegan [35] investigated the following relationship that expresses the cumulative AE energy release during crack extension:

(2) $$\frac{dE}{dN}=\frac{{\left(\Delta K\right)}^{2}D}{E\prime {\left(1-R\right)}^{2}}t\frac{da}{dN},$$

where *E* is the cumulative energy of AE signals; E′ is the elastic modulus under plane stress; *R* is the load ratio; *t* is the specimen thickness and *D* is the constant related to the material properties.

In the crack stable propagation stage, E′ and *D* are constants under the Pairs law [33], while the values of *t* and *R* are constant throughout the experiment. By substituting Equation (1) into Equation (2), the following equation can be obtained:

(3) $$\frac{dE}{dN}=BC^{\frac{-2}{m}}{\left(\frac{da}{dN}\right)}^{\frac{2+m}{m}},$$

where *B* is the constant related to the material properties, with $B=Dt/\left[E\prime {\left(1-R\right)}^{2}\right]$.

Using the logarithms on both sides of Equation (3), the relationship between the AE energy rate and the crack growth rate is obtained as:

(4) $$\ln(\frac{da}{dN})=\alpha \ln(\frac{dE}{dN})+\beta ,$$

where α and β are the constants related to the material properties in the crack stable propagation stage, with α=m/(m+2), $\beta =\ln(C^{2}/B^{m})/(m+2)$.

## 3. Materials and Method

### 3.1. Specimens

**Figure 2:**
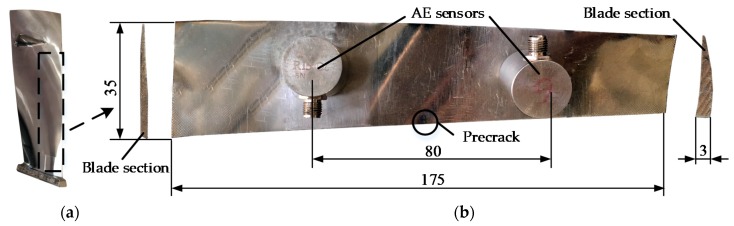
Geometry of gas turbine engine blade specimen (mm): (**a**) gas turbine engine blade; and (**b**) gas turbine engine blade specimen.

**Figure 3:**
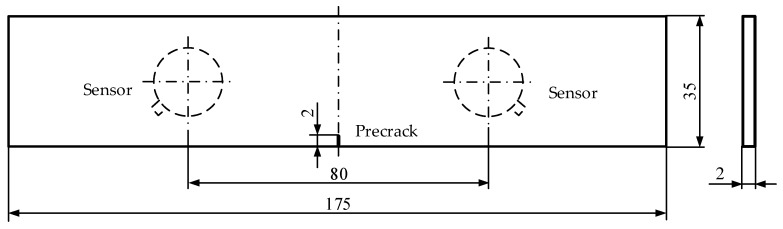
Geometry of the TC11 titanium alloy plate specimen (mm).

The test material was TC11 titanium alloy, which is widely used in gas turbine engine blades [36]. Two types of specimens were used. One specimen was a gas turbine engine low-pressure compressor rotor blade (Figure 2a). In order to facilitate clamping, a portion of the blade was used in the shape shown in Figure 2b. The other specimen consists of three identical TC11 titanium alloy plate samples (refer to ASTM E647) and the geometry of the specimens is displayed in Figure 3. Both specimens were prefabricated with a 2-mm long pre-crack using Electric Discharge Machining (EDM). The alloy composition and main mechanical properties of the TC11 titanium alloy material are shown in Table 1 and Table 2 [36].

### 3.2. Experimental Equipment and Procedures

**Figure 4:**
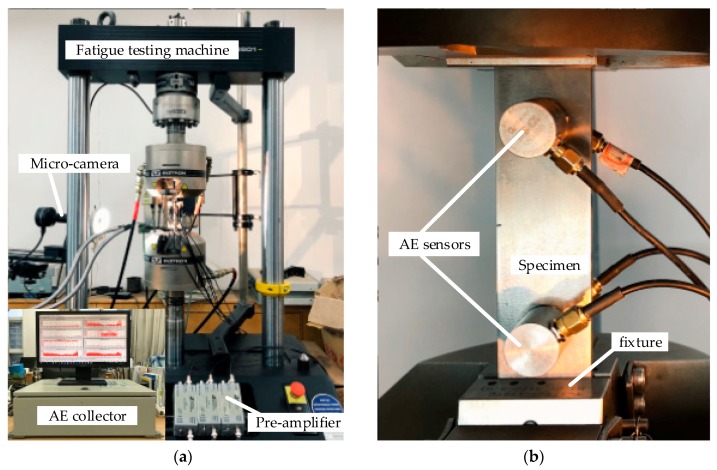
Experimental test system: (**a**) Experimental equipment; and (**b**) Specimen mounted on Instron 8801.

The fatigue crack growth test was performed on an electro-hydraulic mechanical testing machine (Instron 8801 fatigue testing system). The fatigue load was a sinusoidal load with a maximum loading of 10 kN, a minimum loading of 1 kN and a loading frequency of 10 Hz. The surface cracks were measured optically with a high-resolution recording micro-camera (3072 × 2048). The experimental test system is displayed in Figure 4. 

**Figure 5:**
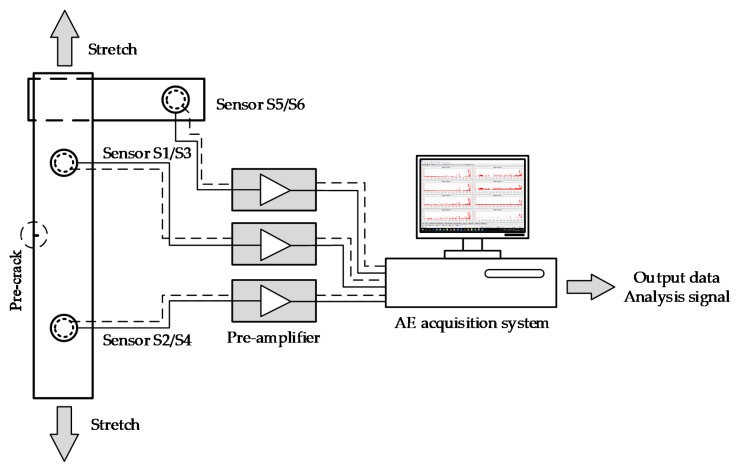
Schematics of the testing system.

The AE signals were collected by a 16-channel AE system, which was comprised of a PC system, pre-amplifiers and six AE sensors. Four sensors were placed on both sides of the specimen. These were placed symmetrically around the pre-crack and were the main sensors used for collecting AE signals. The other two sensors were symmetrically arranged on a TC11 plate, which was in close contact with the specimen and was held by the mechanical testing machine. They were used to acquire the AE signals that passed through the coupling interface. The schematics of the testing system are shown in Figure 5. Table 3 shows the AE software parametric setup and Table 4 shows the relevant information of the AE sensors. The thresholds are the values at which the AE acquisition system failed to collect a signal when the hydraulic system was activated.

## 4. Results and Discussion

### 4.1. AE Signals of TC11 Titanium Alloy

**Figure 6:**
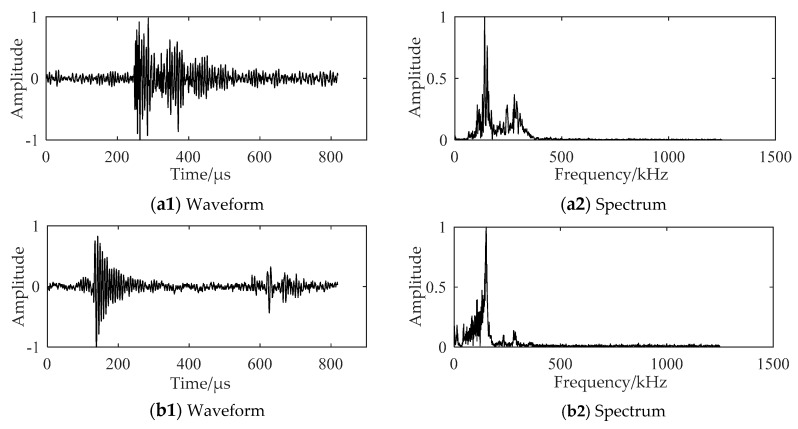
Waveform and spectrum of the AE signals collected from the gas turbine engine blade specimen: (**a**) AE signal of the stable propagation stage (crack length: 2.5 mm); and (**b**) AE signal of the fracture stage (crack length: 17.2 mm) of the crack.

**Figure 7:**
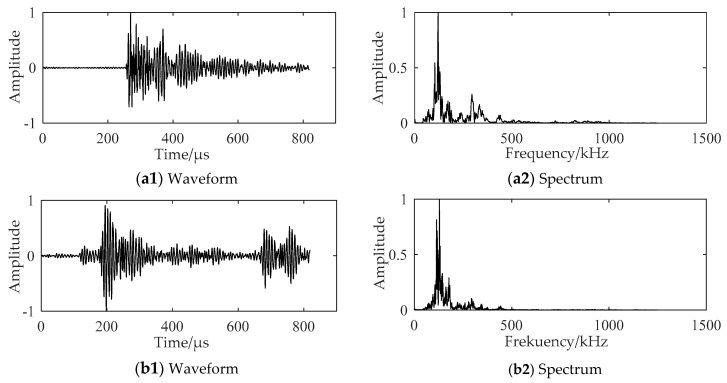
Waveform and spectrum of the AE signals collected from the TC11 titanium alloy plate specimen: (**a**) AE signal of the stable propagation stage (crack length: 2.3 mm); (**b**) AE signal of the fracture stage (crack length: 17.5 mm) of the crack.

The AE signals in the stable propagation stage and the fracture stage of the crack in the gas turbine engine blade specimen are shown in Figure 6(a1,b1), while the frequency spectrums of these stages are plotted in Figure 6(a2,b2). The AE signals of the TC11 titanium alloy plate are shown in Figure 7. The main frequency components of AE signals collected from the blade specimen and the TC11 titanium alloy plate specimen are similar. The AE characteristics of the gas turbine engine blades during the fatigue crack propagation were investigated using the experimental data of the two specimens.

### 4.2. AE Characteristics of Fatigue Crack Growth

**Figure 8:**
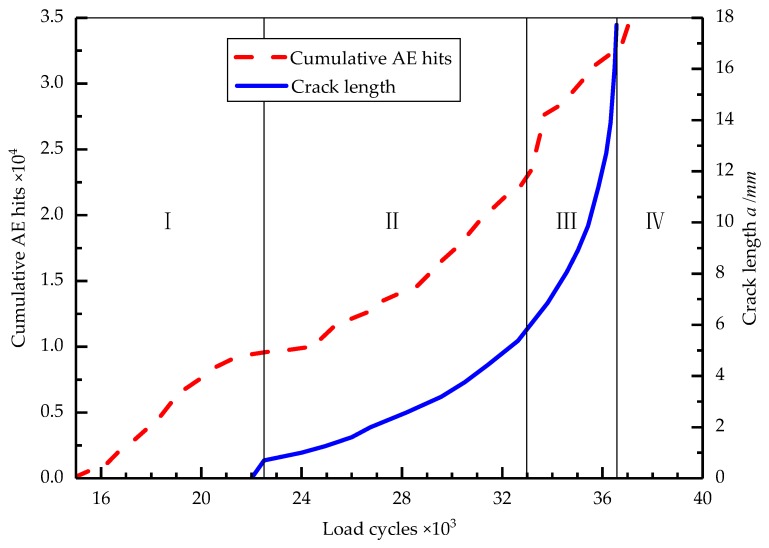
Crack length and cumulative AE hits compared to load cycles.

The crack length and cumulative AE hits compared to the load cycles for the TC11 titanium alloy are displayed in Figure 8. The cumulative AE hits represent the total number of signals received by the AE sensors during the entire monitoring period, which is an important indicator for evaluating the AE activity. It is evident that the curve of the cumulative AE hits exhibits a slow upward trend in the range of 15,000–22,500 cycles (Stage I, Figure 8) and the crack propagation is observed by the micro-camera at around 22,500 cycles, which represents the initiation stage of the crack. The stable propagation stage of the crack occurs in the range of 22,500–33,000 cycles (Stage II, Figure 8) and the number of cumulative AE hits and the crack length exhibit a slow increasing trend during this stage. Starting at 33,000 cycles (Stage III, Figure 8), the slope of the cumulative AE hits curve rapidly increases and the crack growth length curve approaches a straight line at about 36,000 cycles, which represents the unstable propagation stage of the crack. At about 36,800 cycles (Stage IV, Figure 8), the cumulative AE hits curve increases linearly and the specimen fractures completely.

During the process of fatigue crack propagation, the cumulative AE hits and the crack length curves exhibit distinct stages that are similar as the load cycles increase. It is important to note that in the range of 15,000–22,500 cycles, the cumulative AE hits exhibit a clear upward trend, which indicates that a large number of AE events occur in the specimens during this time, although no visible changes were observed by the micro-camera. The AE signals show that the microscopic cracks or crack propagation occurred in the material at that time and they occurred earlier than the macroscopic cracks on the specimen’s surface. Therefore, the trend of the cumulative AE hits reflects the damage of the TC11 titanium alloy material and indicates early fatigue damage in the material.

**Figure 9:**
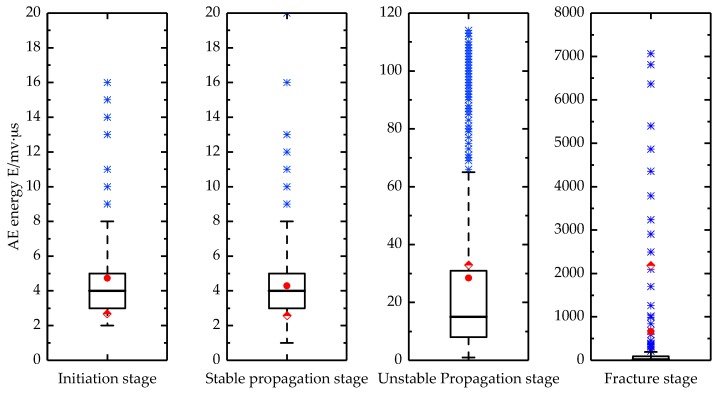
AE energy distribution in the four stages of fatigue crack growth.

**Figure 10:**
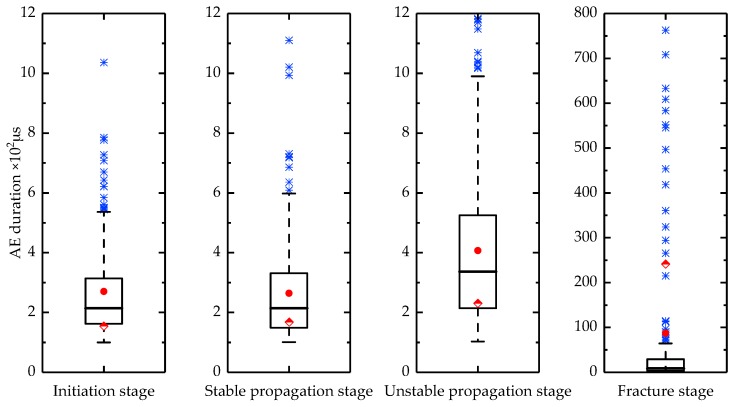
AE duration distribution in the four stages of fatigue crack growth.

AE energy and duration are two important parameters in the analysis of AE signals. The AE energy is the measured area under the rectified signal envelope, which reflects the relative energy of the event. The AE duration represents the time that the waveform amplitude remains above the threshold and it is commonly used for the identification of particular AE sources. Therefore, the energy and duration of the AE signals during the four stages of crack propagation were statistically analyzed in this present study and were illustrated by box-plots. As shown in Figure 9 and Figure 10, where Stage I represents an intermediate stage, Stage II represents a stable propagation stage, Stage III represents an unstable propagation stage and Stage IV represent a fracture stage. The top line represents the maximum value, while the bottom line is the minimum value, and the blue points outside these two lines are outliers. The bottom and top lines of the box represent the 25th and the 75th percentile, while the bold line inside the box indicates the median. The rounded and diamond points represent the mean and standard deviation, respectively, for the data.

The energy and duration of the AE signals exhibit similar patterns during the initiation and stable propagation stages of the crack. The major source of the AE signals in these two stages is associated with the dislocation and slip activity in the grain boundaries [37,38]. During the unstable propagation stage, the AE energy and duration increase, while the ranges also increase. This is partly attributed to the increase in the signal intensity of the cracks, while more high-energy signals are derived from the friction and impact generated by the closure of the crack during the low-stress phase. At the moment of the blade fracture, a large number of AE signals are generated and some of the signals have energies and durations that are 2–3 orders of magnitude larger than the energies and durations during the first three stages. The transition point between the stable and unstable cracking is referred to as the critical cracking level [39]. Therefore, the AE energy and duration during the four stages allow us to identify when the blade cracks enter the unstable propagation stage, which will allow us to take timely measures to prevent further damage caused by the blade fracture.

### 4.3. Fatigue Life Prediction

**Figure 11:**
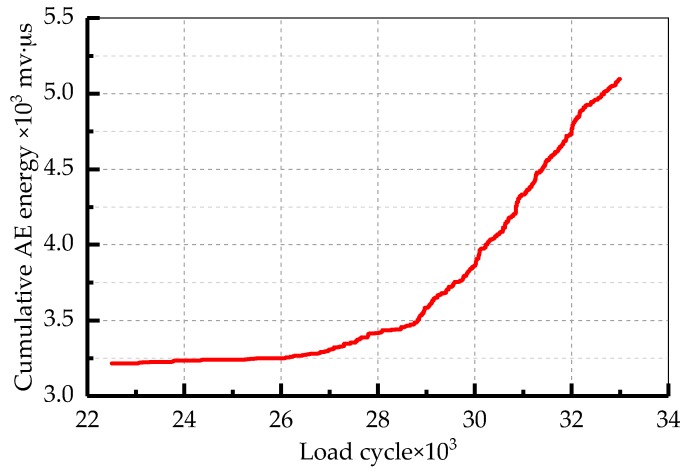
Cumulative AE energy compared to load cycles in Stage II.

**Figure 12:**
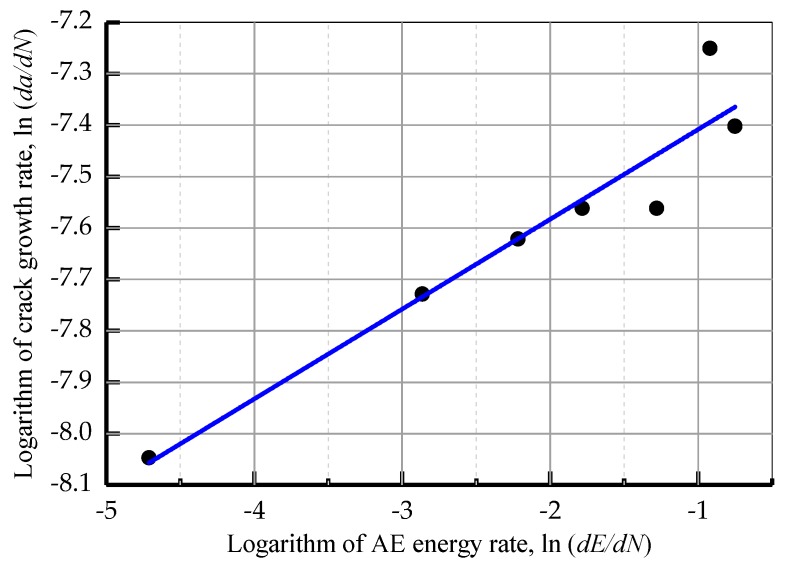
Crack growth rate compared to AE energy rate in Stage II.

The cumulative AE energy compared to the load cycles during the crack stable propagation stage (Stage II) for the TC11 titanium alloy are displayed in Figure 11. The cumulative AE energy shows a regular upward trend. According to Equation (4), there is a linear relationship between the logarithms of the AE energy rate and the crack growth rate. Therefore, the least squares method was used to linearly fit the values of the two parameters (Figure 12). The values of the constants were obtained during the stable propagation stage of the crack and the correlation coefficient was 0.946. The relationship between the AE energy rate and crack growth rate is:

(5) $$\ln(\frac{da}{dN})=0.175\ln(\frac{dE}{dN})-7.234.$$

**Figure 13:**
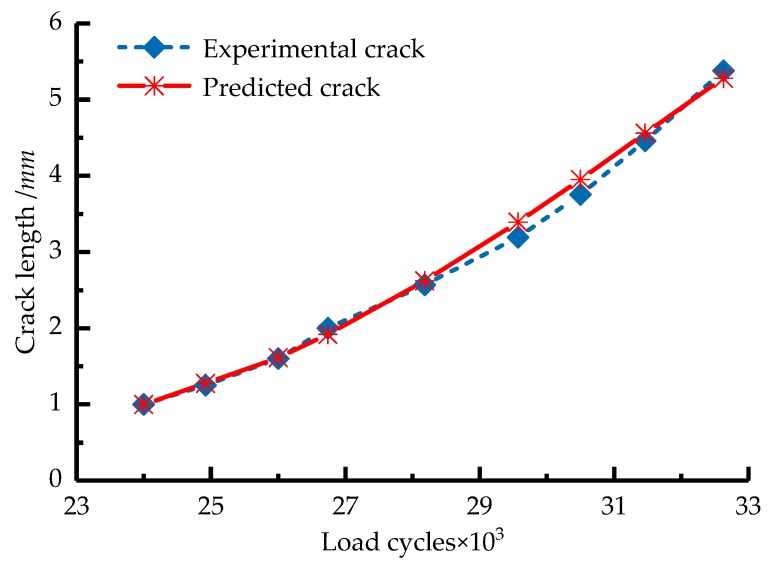
Predicted and actual crack length in Stage II.

Based on Equation (5), the crack length during the crack stable propagation stage (Stage II) was predicted and compared with the experimental crack length (Figure 13). The predicted crack length was consistent with the experimental crack length. Therefore, we can use the measured AE energy rate to calculate the crack growth rate and to predict the crack growth state and residual fatigue life of the blade. Furthermore, compared with Equation (1), this method does not require the calculation of the stress intensity range, which is difficult to accurately measure under actual working conditions.

## 5. Conclusions

In this present study, we propose the use of AE monitoring technology for the online identification of the blade state. AE-based fatigue monitoring tests were performed on the gas turbine engine blade and TC11 titanium alloy plate specimens. The relationship between the cumulative AE hits and the fatigue crack length was established, before a method of using AE parameters to determine the crack propagation stage was proposed. A crack growth prediction methodology based on the AE signal energy was presented and validated. Furthermore, a model relating the AE energy rate to the crack growth rate was presented for predicting the crack extension and remaining fatigue life for gas turbine engine blades. This model can avoid the calculation of the stress intensity range. The following conclusions can be drawn:

1. The use of AE monitoring technology can be used for the timely detection of the growth stages of fatigue cracks. The relationship between the cumulative AE hits and the number of cycles indicated the same variation as observed by the micro-camera of the crack propagation. However, the AE technique is more sensitive and can detect the initiation of the cracks in the materials to achieve early identification of fatigue damage.
2. The characteristics of the AE parameters, such as energy and duration, provide warning signs for gas turbine engine blades at the critical point where the fatigue cracks enter the stage of unstable propagation, which ultimately leads to catastrophic failure.
3. The proposed mathematical model of the relationship between AE energy rate and crack growth rate provides a basis for determining the crack propagation state and predicting the remaining fatigue life of gas turbine engine blades. Specific material constants should be evaluated through testing prior to the implementation of the proposed model.

## Figures and Tables

**Table 1:** Compositions of the TC11 titanium alloy.

Alloy Element	Al	Mo	Zr	Si	Ti
Mass fraction (%)	5.8–7.0	2.8–3.8	0.8–2.0	0.20–0.35	Margin

**Table 2:** Main mechanical properties of the TC11 titanium alloy (room temperature).

$\sigma_{b}/\mathrm{MPa}$	$\sigma_{p0.2}/\mathrm{MPa}$	$\delta_{5}/\%$	ψ/%	$a_{k\upsilon }/({\mathrm{kJ}/m}^{2})$
1030–1225	930	9	30	295

Note: $\sigma_{b}$ is the tensile strength, $\sigma_{p0.2}$ is the specified non-proportional elongation strength, $\delta_{5}$ is the elongation after failure, ψ is the reduction of area and $a_{k\upsilon }$ is the impact toughness of the V-notch specimen.

**Table 3:** AE software parametric setup.

Parameter	Value
Sample Rate/MS/s	2.5
Hit Length/K	8
Peak Definition Time/μs	300
Hit Definition Time/μs	600
Hit Lockout Time/μs	1000

**Table 4:** AE sensor information.

Sensor Number	Resonant Frequency/kHz	Operating Freq. Range/kHz	Threshold/dB
S1/S2	150	50–200	50
S3/S4	125	100–1000	40
S5	150	50–200	45
S6	125	100–1000	35
